# Role of household factors in parental attitudes to pandemic influenza-related school closure in Japan: a cross-sectional study

**DOI:** 10.1186/1471-2458-14-1089

**Published:** 2014-10-21

**Authors:** Mitsuo Uchida, Minoru Kaneko, Shigeyuki Kawa

**Affiliations:** Center for Health, Safety and Environmental Management, Shinshu University, 3-1-1 Asahi, Matsumoto, Nagano, 390-8621 Japan

**Keywords:** Influenza, Pandemic, School closure, Household, Attitude

## Abstract

**Background:**

To investigate how household background factors affect parental behavior during pandemic influenza-related school closures, we determined associations between such factors and three parental attitudes: “caring for the child”, “taking leave from work”, and “permitting out-of-home activities”.

**Methods:**

A hypothetical pandemic influenza situation was presented and a questionnaire survey among households of 2146 schoolchildren from 6 schools was conducted. Odds ratios of background factors were estimated using univariate and multivariate logistic regression models.

**Results:**

Responses pertaining to 1510 children indicated that junior high school (OR = 0.11), both parents working (OR = 0.03), and family including grandparent(s) or other relatives (OR = 7.50) were factors associated with “caring for the child”, and elementary school (OR = 2.28), special education school (OR = 3.18), and both parents working (OR = 5.74) were associated with “taking leave from work”. Having an older sibling (OR = 0.74) and awareness of the technical term for school closure (OR = 0.73) were factors associated with “permitting out-of-home activities”.

**Conclusion:**

Not only work status but also other household factors may be associated with parental behaviors during pandemic influenza-related school closures.

## Background

School closure is a non-pharmaceutical intervention for infection control. School closure is known to include “proactive closure” and “reactive closure”, the former of which is thought to be especially effective in suppressing virus transmission [1]. In Japan, the government required schools to be closed for approximately one week simultaneously at the beginning of the 2009 influenza (H1N1) pandemic [2]. Many schools in several other countries were also closed to minimize spread of the virus [3, 4]. Although school closures were thought to limit the spread and transmission of infection, the optimal durations and timings of school closure remain unclear [3, 4]. As school closure is affected by several environmental and individual factors, these associations should be clarified.

Parental and student behavior are thought to have affected the effectiveness of school closure during the 2009 influenza (H1N1) pandemic [2]. Several studies [5–10] investigated the behavior of students and parents during school closure and reported that some students had engaged in out-of-home activities and some parents had taken leave from work in 2009. The proportions of these behaviors varied among reports, and the factors underlying the variations in parental and student behavior remain unclear. It is important to clarify which factors influence parental and student behavior to evaluate the effectiveness of school closure as an infection control measure.

Here, we conducted a cross-sectional questionnaire survey to clarify the associations between household background factors and parental behaviors. A hypothetical pandemic influenza situation was presented with questions about parental attitudes and probable behaviors, including “caring for the child”, “taking leave from work”, and “permitting out-of-home activities”.

## Methods

### Study population

Subjects comprised households of schoolchildren attending six schools (one kindergarten, two elementary schools, two junior high schools, and one special educational needs school) all attached to Shinshu University, Nagano, Japan. Because these six schools had been investigated continuously for infectious disease epidemiology [11, 12], this study was conducted in these same schools. The ages of children attending kindergarten, elementary school, junior high school and special education needs school were 4–6, 7–12, 13–15 and 7–18 years old, respectively. Households of 2146 children attending these six schools in July 2013 received one questionnaire per child from their teacher. Questionnaires were answered anonymously by parents or guardians and returned to teachers in a sealed envelope. The study design and procedure were reviewed and approved by the Committee for Medical Ethics of Shinshu University (approval number 2327).

### Measures and variables

The following hypothetical situation was presented in the questionnaire and responses were elicited: “A pandemic influenza outbreak has arisen resulting in school closure but your child is not infected. Please indicate your probable behaviors during the period of school closure”. The attitude items included those examined in previous reports [6–10]: “caring for the child”, i.e., the parent expressed that they would actively take care of their child in the event of school closure; “taking leave from work”, i.e., the parent expressed that they would take time off work in the event of school closure; and “permitting out-of-home activities”, i.e., the parent expressed that they would not confine their child to their home in the event of school closure. In addition, the household factors included family structure and ages (description), regular employment of each household member (yes or no), awareness of the technical term for school closure (yes or no), and acceptable duration of school closure (description). In this questionnaire, awareness of the technical term for school closure means knowledge of the two types of school closure; namely proactive closure and reactive closure. The former is applied at the beginning of an epidemic to prevent transmission among children and the latter is applied if many children or staff are absent because of illness so classes cannot be held [1]. Thus, the purpose of these two closure types is quite different. In this study, households including a member who was aware of these technical terms for the two types of closure were considered to be aware of the purpose of school closure.

### Statistical analysis

Responses concerning the two elementary schools were grouped together and those of the two junior high schools were grouped together then totaled as elementary and junior high school respectively because they are administrated under the same rules. Hence, school affiliations were divided into four types (kindergarten, elementary school, junior high school, and special educational needs school). The other categorical factors were divided into two contingencies. The chi-square test was used for comparison of differences among categories, and the Mann–Whitney test and Kruskal–Wallis test were used to compare continuous variables. Univariate and multivariate logistic regression models were used to estimate the odds ratios (OR) and 95% confidence intervals (95%CI). All analyses were performed using SPSS ver.22 for Windows (SPSS, Chicago, IL), and *P* <0.05 was taken to indicate statistical significance.

## Results

Questionnaires were returned by the parents of 1711 of the 2146 children (response rate: 79.7%) and 1510 were included in the analysis after excluding 201 with incomplete responses. A comparison of characteristics between complete and incomplete responders revealed some differences. The rate of complete responses was significantly lower in “special education needs school (*P* =0.036)” in school affiliation factor and in “other than both working (*P* <0.001)” in type of work factor, however, other factors showed no differences. Of these complete answers, 1485 households (98.3%) had work activity, and both parents were working in 921 (61.0%) households. Table 1 shows the associations between the household background factors and three attitudes and acceptable duration of school closure. The attitude “caring for the child” was reported in 1118 (74.0%) households and was significantly different among school affiliations. We checked the residuals to compare the frequency of answers and “yes” responses were significantly greater in kindergarten and elementary schools. In addition, this attitude was also significantly associated with type of work activity and family structure. The attitude “taking leave from work” was reported in 507 (33.6%) households and was significantly different among school affiliations. We also checked the residuals to compare the frequency of answers and “yes” responses were significantly prominent in elementary schools. In addition, it was also associated with the type of work activity. The attitude “permitting out-of-home activities” was reported in 943 (62.5%) households and was associated with school affiliation and having an older sibling. Although not statistically significant (*P* =0.051), awareness of the technical term for school closure tended to be associated with this attitude. The acceptable duration of school closure differed according to type of work activity, family structure, and awareness of the technical term for school closure.

**Table 1:** **Associations between household background factors and parental attitudes**

Household background factors		Responses	Care for child					Leave from work					Permit out-of-home activity					Acceptable duration of school closure (days)			
		(n = 1510)	No	(%)	Yes	(%)	***P***	No	(%)	Yes	(%)	***P***	No	(%)	Yes	(%)	***P***	Median	25%	75%	***P***#
			392		1118			1003		507			567		943						
School affiliation																					
	Kindergarten	68	1	(1.5)	67	(98.5)	<0.001	60	(88.2)	8	(11.8)	<0.001	22	(32.4)	46	(67.6)	0.013	5	3	7	0.183
	Elementary school	723	137	(18.9)	586	(81.1)		458	(63.3)	265	(36.7)		247	(34.2)	476	(65.8)		4	3	5	
	Junior high school	686	249	(36.3)	437	(63.7)		468	(68.2)	218	(31.8)		288	(42.0)	398	(58.0)		4	3	5	
	Special education needs school	33	5	(15.2)	28	(84.8)		17	(51.5)	16	(48.5)		10	(30.3)	23	(69.7)		3	2	5	
Type of work activity																					
	Other than both working	589	11	(1.9)	578	(98.1)	<0.001	507	(86.1)	82	(13.9)	<0.001	210	(35.7)	379	(64.3)	0.224	5	3	7	<0.001
	Both working*	921	381	(41.4)	540	(58.6)		496	(53.9)	425	(46.1)		357	(38.8)	564	(61.2)		3	3	5	
Family structure																					
	Nuclear family	1297	373	(28.8)	924	(71.2)	<0.001	872	(67.2)	425	(32.8)	0.101	476	(36.7)	821	(63.3)	0.093	4	3	5	0.037
	Family including grandparent or others	213	19	(8.9)	194	(91.1)		131	(61.5)	82	(38.5)		91	(42.7)	122	(57.3)		5	3	7	
Having older sibling																					
	No	919	229	(24.9)	690	(75.1)	0.250	604	(65.7)	315	(34.3)	0.472	321	(34.9)	598	(65.1)	0.009	4	3	6	0.331
	Yes	591	163	(27.6)	428	(72.4)		399	(67.5)	192	(32.5)		246	(41.6)	345	(58.4)		4	3	5	
Awareness of technical term for school closure																					
	No	1346	352	(26.2)	994	(73.8)	0.627	901	(66.9)	445	(33.1)	0.225	494	(36.7)	852	(63.3)	0.051	4	3	5	0.027
	Yes	164	40	(24.4)	124	(75.6)		102	(62.2)	62	(37.8)		73	(44.5)	91	(55.5)		5	3	7	

*Including single parents who were working.

#Kruskal-Wallis test and Mann–Whitney test were used.

To evaluate the effect of household background factor for parental attitude, odds ratios of factors were determined by using logistic regression analysis. Moreover, several factors showed significant effects simultaneously, multivariate analysis was used to adjust for background factors (Table 2). As no significant internal correlations were found between variables, all variables were used in multivariate analysis. As a result, the proportion of responses to “caring for child” was lower in households with children in junior high school (OR =0.11, 95% CI 0.01-0.86, *P* =0.035), but higher in “family including grandparent(s) or other relatives” (OR =7.50, 95% CI 4.53-12.44, *P* <0.001). “Leave from work” was higher in families with children in elementary school (OR =2.28, 95% CI 1.03-5.01, *P* =0.041), special educational needs school (OR =3.18, 95% CI 1.10-9.22, *P* =0.033), and with “both parents working” (OR =5.74, 95% CI 4.33-7.60, *P* <0.001). The rate of “permitting out-of-home activities” was significantly lower in families responding “yes” to “having older siblings” (OR =0.74, 95% CI 0.59-0.91, *P* =0.005) and tended to be lower in those with “awareness of the technical term for school closure” (OR =0.73, 95% CI 0.52-1.01, *P* =0.056).

**Table 2:** **Effects of household factors on parental attitudes**

Household background factors		Responses	Care for child								Leave from work								Permit out-of-home activity							
		(n = 1510)	Univariate model				Multivariate model				Univariate model				Multivariate model				Univariate model				Multivariate model			
			OR	95% CI		***P***	OR	95% CI		***P***	OR	95% CI		***P***	OR	95% CI		***P***	OR	95% CI		***P***	OR	95% CI		***P***
School affiliation																										
	Kindergarten	68	1.00				1.00				1.00				1.00				1.00				1.00			
	Elementary school	723	0.06	0.01	0.46	0.007	0.23	0.03	1.80	0.160	4.34	2.04	9.22	<0.001	2.28	1.03	5.01	0.041	0.92	0.54	1.57	0.763	0.95	0.55	1.62	0.839
	Junior high school	686	0.03	0.01	0.19	<0.001	0.11	0.01	0.86	0.035	3.49	1.64	7.43	0.001	1.35	0.61	2.99	0.464	0.66	0.39	1.12	0.126	0.66	0.38	1.15	0.141
	Special education needs school	33	0.08	0.01	0.75	0.026	0.27	0.03	2.76	0.271	7.06	2.58	19.29	<0.001	3.18	1.10	9.22	0.033	1.10	0.45	2.70	0.836	1.13	0.45	2.81	0.800
Type of work activity																										
	Other than both working	589	1.00				1.00				1.00				1.00				1.00				1.00			
	Both working*	921	0.03	0.02	0.05	<0.001	0.03	0.01	0.05	<0.001	5.30	4.06	6.92	<0.001	5.74	4.33	7.60	<0.001	0.88	0.71	1.09	0.224	0.98	0.78	1.23	0.856
Family structure																										
	Nuclear family	1297	1.00				1.00				1.00				1.00				1.00				1.00			
	Family including grandparent or others	213	4.12	2.54	6.70	<0.001	7.50	4.53	12.44	<0.001	1.28	0.95	1.73	0.101	0.90	0.65	1.24	0.516	0.78	0.58	1.04	0.093	0.78	0.58	1.06	0.109
Having older sibling																										
	No	919	1.00				1.00				1.00				1.00				1.00				1.00			
	Yes	591	0.87	0.69	1.10	0.250	0.78	0.59	1.03	0.079	0.92	0.74	1.15	0.473	0.83	0.66	1.05	0.122	0.75	0.61	0.93	0.009	0.74	0.59	0.91	0.005
Awareness of technical term for school closure																										
	No	1346	1.00				1.00				1.00				1.00				1.00				1.00			
	Yes	164	1.10	0.75	1.60	0.627	1.35	0.87	2.08	0.182	1.23	0.88	1.72	0.225	1.20	0.84	1.73	0.313	0.72	0.52	1.00	0.052	0.73	0.52	1.01	0.056

*Including single parents who were working.

Multivariate model: variables were adjusted for each other in the multivariate logistic regression model.

## Discussion

We conducted a cross-sectional attitude questionnaire survey among households of children attending six schools to clarify the associations between household background factors and parental behavior during periods of school closure as an infection control measure. We found that the attitudes “caring for children”, “taking leave from work”, and “permitting out-of-home activities” were associated with various household background factors.

The attitude of caring for children was reported by 74.0% of households in this study, which was similar to the findings of a study [10] that reported daily care of children by parents during school closure due to H1N1 in 71.6% of households in Japan. The level of caring attitude differed among school affiliations (effectively the grade of school) in this study, in contrast to a study in the USA, which showed that the main caregivers during school closure were the students themselves, or parents with no clear trend by grade [8]. Thus, the association between the attitude of parents and grade of children may differ among countries or cultures. In addition to this result, the attitude of caring for children by parents was associated with family structure. Although the behavior of caring for children during school closure was generally thought to be dependent only on the employment status of the parents, which was also observed in this study, we found that the inclusion of grandparent(s) or other family members in the household was associated with “caring for the child” by the parents. Although this result was natural, the factor of family structure has not been taken into consideration in infection control measures. Therefore, if households are required to care for their children during periods of school closure due to pandemic influenza, future measures or guidelines should include reference to not only work activity but also household member availability.

“Taking leave from work” during periods of school closure was reported by 33.6% of households. A report showed that members from 12.9% of households took leave from work during pandemic influenza-related school closures in Japan [10], which was much lower than the finding in the present study. This disparity may have been because our study included households of children attending kindergarten to junior high school, whereas the previous study included high school. In addition to this disparity, in other countries, the rates of a parent taking leave from work during school closures varied widely: 17% at seven schools in New York City [9], 20% in 39 states in the USA [5], and 45% at three schools in Perth, Australia [6]. Therefore, we supposed that the variation may be affected by some other household factors. Both parents working was associated with taking leave from work during school closure in this study, so this factor may affect such variation. In addition, in this study, having children in elementary schools and special education needs schools showed stronger associations with taking leave from work during school closure than having children in kindergarten. This disparity may have been because the parents of kindergarten-age children mulled over the decision to leave their children in a daycare center in the event of a pandemic. Therefore, more information may have been acquired if this study had included a daycare center, and this issue should be clarified in future. In general, the proportion of households where a parent said they would take leave from work due to school closure is important information for the development of a Business Continuity Plan (BCP) [2]. As it is difficult to manage both the BCP and school closure measures simultaneously and further information, such as “school affiliation” or “type of work activity” should be obtained and both BCP and school closure measures should be reconciled for determination of practical measures in future.

There were cases where children spent time outside of their homes during pandemic influenza-related school closure and made contact with each other [2] resulting in virus transmission weakening the effectiveness of infection control measures. The parental behavior of permitting out-of-home activities during school closure occurred during the influenza (H1N1) 2009 pandemic and varied by country, with rates of 20.5% in Japan [10], 34% [9] and 69% [7] in the USA, and 75% [6] in Australia. The behavior also differed according to school grade or day of the week [8]. Therefore, it is important to determine that factors associated with these variations. In this study, we investigated the associations between household background factors and attitudes, and found that “having an older sibling” was associated with not permitting out-of-home activity. This was thought to be because whether children have a sibling with whom to play during school closure was associated with the parents’ attitude toward permitting out-of-home activities. Therefore, “having an older sibling” was an important factor in controlling children’s behavior, and at least indication of own behavior (e.g., homework or private study) during school closure may suppress out-of-home activity. Lack of awareness of the technical term for school closure tended to be associated with “permitting out-of-home activities”. A previous report showed that knowledge of H1N1 transmission pattern was associated with hygiene improvement [13]. We assume that subjects with knowledge of influenza characteristics might improve their infection control measures. In the present study, because subjects with awareness of technical term of school closure were regarded as knowledgeable about the purpose of the closures and characteristics of influenza, we speculate that this knowledge influenced parental behavior of forbidding out-of-home activity. Thus, explicit explanation of the aims of school closure may influence behavior and improve the effectiveness of this measure for infection control.

This study had several limitations. First, actual behavior during school closure due to the influenza pandemic may differ from that reported by respondents as attitudes may not always reflect actions. However, families in Japan are accustomed to school closures during seasonal influenza outbreaks and have most likely all experienced the behaviors being discussed. Therefore, this limitation is likely very minimal in this particular study conducted among Japanese culture. Second, household behaviors “parental work activity” and “permitting out-of-home activities” may have differed according to schools or districts [9] and responses among parents of children attending the same school may have been more similar to each other than to those of parents of children attending another school. Although multivariate analysis was used in analysis, this clustering effect might have remained. However, the present study was based on a hypothetical situation and included only 6 schools, therefore we regarded that detailed adjustment of clustering may not be important. Further data of more schools should be accumulated to clarify any such possible clustering effect in a future study. Third, the rate of complete answers were biased in school affiliation and type of work factors. This phenomenon may reflect the interests of the study population in infection control measures. This disparity may slightly affect the study results. Fourth, behavior may change according to the pathogenicity of the influenza virus. As the pathogenicity of the influenza (H1N1) 2009 virus was not as strong as expected before the pandemic, on which they may have based their responses due to their memory of this event, the respondents’ impressions of the pandemic influenza virus may have been underestimated. However, an influenza virus with high pathogenicity will likely induce an influenza pandemic in the future. When pandemic influenza occurs in the future, it will be important to distribute up-to-date information regarding the pathogenicity of the virus immediately and previous experience of influenza H1N1 (2009) should not be an inhibiting factor.

## Conclusion

In this study, we determined associations between household background factors and parental attitudes describing their likely behavior during periods of pandemic influenza-related school closure for infection control. We found that factors of school affiliation, family structure, type of work activity, and knowledge of the technical term for school closure were associated with parental attitudes. This additional information will be useful for future infection control measures, including school closure.
